# A systematic review of the efficacy of a single dose artemisinin–naphthoquine in treating uncomplicated malaria

**DOI:** 10.1186/s12936-015-0919-5

**Published:** 2015-10-06

**Authors:** Cho Naing, Maxine A. Whittaker, Joon Wah Mak, Kyan Aung

**Affiliations:** Institute for Research, Development and Innovation, International Medical University, Kuala Lumpur, Malaysia; School of Public Health, University of Queensland, Brisbane, Australia; School of Medicine, International Medical University, Kuala Lumpur, Malaysia

**Keywords:** Artemisinin–naphthoquine, Malaria, Randomized controlled trials, Systematic review

## Abstract

**Background:**

This study aimed to synthesize the existing evidence on the efficacy and safety of a single dose artemisinin–naphthoquine (ASNQ) for treatment of uncomplicated malaria in endemic countries.

**Methods:**

A meta-analysis of randomized, controlled trials (RCT), assessing efficacy and safety of single dose ASNQ was carried out. Comparator drugs included artemether–lumefentrine (AL), chloroquine plus sulfadoxine-pyrimethamine (CQSP) and dihydroartemisinin–piperaquine (DHP). The efficacy and safety profile of non-comparator, single-arm studies on the single dose ASNQ was also assessed. The primary endpoint was efficacy defined as an absence of PCR-confirmed parasitaemia. The methodological quality of the included studies was assessed using the six domains for the risk of bias.

**Results:**

Five RCTs and three single-arm studies were included in this review. As RCT studies did not compare the same anti-malarial drugs, it was difficult to do a pooled analysis. At day 28, a pooled analysis of two RCTs (n = 271) showed a comparable efficacy on PCR-confirmed parasitaemia between ASNQ and AL. Another RCT, which compared ASNQ and CQSP or ASNQ and DHP, also showed comparable efficacy. At day 42, one RCT comparing ASNQ and DHP and another RCT comparing ASNQ and AL reported comparable levels of efficacy. The proportion of parasite clearance was faster in the ASNQ groups than the comparators at day 1, and almost all parasites were cleared by day 3 in the ASNQ groups.

**Conclusions:**

The present review provides some evidence to support that there is similar efficacy and safety of the single dose ASNQ compared to other anti-malarial drugs in treating uncomplicated malaria. Larger, adequately powered, well-designed studies are recommended to substantiate the efficacy and safety in different populations and in different epidemiological settings. As the potential evolution of drug resistance is a great concern and this cannot be addressed in a short-term study, the use of single dose ASNQ needs further evaluation.

**Electronic supplementary material:**

The online version of this article (doi:10.1186/s12936-015-0919-5) contains supplementary material, which is available to authorized users.

## Background

The target set under Millennium Development Goal 6 will be reached by 55 countries that are on track to reduce their malaria burden by 75 % [1]. Despite progress in the reduction of malaria morbidity and mortality in recent years, malaria remains one of the leading health problems in endemic countries. It has been estimated that there were 198 million (124–283 million) cases of malaria and 584,000 (136,000–755,000) deaths from malaria worldwide in 2013. The vast majority of cases (90 %) occurred in the African region [1]. Being a curable disease, early diagnosis and prompt treatment is a key strategy to reduce morbidity and mortality from malaria. A core component of any malaria elimination programme is to ensure that all patients with malaria are rapidly diagnosed, have access to highly effective anti-malarial drugs and are able to complete the course of treatment [2].

The development of resistance to conventionally used anti-malarial drugs, such as chloroquine (CQ) and sulfadoxine-pyrimethamine (SP) has been documented [3]. WHO recommended that artemisinin-based combination therapy (ACT) should be used for treating uncomplicated *Plasmodium falciparum* malaria to ensure efficacy and reduce the emergence of drug-resistant parasites [1, 4, 5]. Since 2007, WHO had recommended that oral artemisinin monotherapy should be gradually phased out and replaced with ACT [1]. The concept of combination therapy relies on the rapid onset of schizonticidal action [6, 7] to rapidly reduce parasitaemia, leaving the residual parasitaemia to be cleared by high concentrations of the partner drug [8].

Resistance of malaria parasites to currently used ACT has emerged and is following a similar pattern of resistance previously observed with other anti-malarial drugs. Thus far, studies have documented evidence of *P. falciparum* resistance to artemisinin (the key component of all ACT) in five countries of the Greater Mekong Sub-region, such as Cambodia, Laos, Myanmar, Thailand, and Vietnam [1] and this was confirmed on the Cambodia-Thailand border [4]. Thus, it is crucial to monitor the efficacy and safety of newly formulated ACT in view of artemisinin resistance.

A recent development of an oral single dose ACT therapy is a coformulated combination of artemisinin and naphthoquine phosphate (NQ) [6, 7, 9]. NQ is absorbed rapidly and completely after oral administration, and reaches peak plasma concentration 2–4 h after administration [6, 10, 11]. It has a long elimination half-life greater than 255 h [12]. Its partner drug, artemisinin is a short-acting drug with elimination half-life of 0.87 (±SD 0.23) h [13]. With due attention to evidence of artemisinin resistance, artemisinin–naphthoquine (ASNQ) and any ACT which has a component of artemisinin are subject to concerns on rational use and efficacy.

Individual studies assessing efficacy and safety of the single dose ASNQ are available. As transmission of malaria varies even over small distances [14], information from clinical studies across endemic countries is of immense value. Thus, it is deemed worthwhile to aggregate the efficacy and safety of single dose ASNQ compared to commonly (and currently) used anti-malarial regimens. Taken together, the objective of the present study was to synthesize existing evidence on the efficacy and safety of single dose ASNQ for treatment of uncomplicated malaria in endemic countries.

## Methods

The present study adhered to the preferred reporting items for systematic reviews and meta-analyses (PRISMA) [15]. The standard methods of systematic review of clinical studies as described in the Cochrane Systematic Review Handbook were applied [16].

### Study search

Studies on the assessment of efficacy of single dose ASNQ in treating uncomplicated malaria were searched in electronic databases such as MEDLINE, EMBASE, CINHAL and the Cochrane Library. For ongoing and unpublished trials, the websites of WHO, the clinical trials database, and the drug manufacturer were checked. The reference sections of the selected studies and relevant reviews were also checked for the possibility of any additional papers. The search was limited to human studies, published in English, French and Chinese languages until May 2015. Medical subject headings (MeSH) terms and text words were (artemisinin–naphthoquine OR ARCO) AND (treatment success OR treatment failure OR efficacy OR tolerability OR safety) AND (malaria OR uncomplicated malaria OR asymptomatic malaria OR *Plasmodium* OR *falciparum* OR *vivax*).

### Study selection

Studies following the PICOS criteria [15] were included.

*Participants* (P) Participants residing in endemic countries and having uncomplicated malaria, regardless of age, gender, pregnancy status, and species of malaria parasite were considered. Diagnosis of malaria was based on microscopy of Giemsa-stained peripheral blood films or a rapid-onsite diagnostic test. Subsequent PCR-based analysis for species confirmation was an additional merit.

*Intervention* (I) Studies using fixed combination single dose ASNQ by participants in one arm, regardless of route of administration and brand name were considered.

*Comparison* (C) Studies which compared the efficacy of single dose ASNQ to alternative anti-malarial drug(s) or placebo were included.

*Outcomes* (O) For simplicity sake, the primary and secondary outcomes were efficacy and safety, respectively. Efficacy was defined as the proportion of absence of (1) PCR-confirmed parasitaemia in patients at day 28 and at day 42, (2) PCR-unconfirmed parasitaemia in patients at day 28 and at day 42, or, (3) parasitological and fever clearance time. The safety outcomes were incidence of adverse events (AEs) and serious adverse events (SAEs). Studies were included if the effect estimates of each study such as relative risk (RR), odds ratio (OR) or hazards ratio (HR) and its 95 % confidence interval (CI) were provided or made available for computation.

*Study design* (S) Randomized controlled trials (RCTs) which assessed efficacy of fixed-combination single dose ASNQ in treating uncomplicated malaria were included. Information from single-arm trials were also considered separately for the therapeutic efficacy of ASNQ, although they were not included in the main meta-analysis. Abstracts and conference reports were included, if they provided adequate data on the comparable efficacy between single dose ASNQ and other anti-malarial drug(s).

### Data extraction

Two authors independently screened the title and abstract yielded from the electronic search. Any discrepancies between the two authors were resolved by consensus. The two authors individually collected information from each included study using the piloted data extraction form. Information collected included characteristics of participants, study design, characteristics of the experimental drug (dosage, route of administration, brand of ASNQ), confirmation of malaria infection, specific speciation, duration of follow-up and the reported clinical outcomes. For studies with overlapping of study population, the one that provided the most comprehensive data was used.

The methodological quality of the included studies was assessed using the risk of bias tool applied for the Cochrane systematic reviews [16]. The six domains for the risk of bias: random sequence generation, allocation concealment, blinding of outcome assessment, incomplete outcome data adequately addressed, free of suggestion of selective outcome reporting and other sources of potential bias addressed. Any discrepancies between the two authors were resolved by discussion. Analysis was done on an intention-to-treat (ITT) basis, whenever possible.

### Statistical analysis

RR and its 95 % CI for dichotomous data and/or mean difference (MD) and standard deviation (SD) for continuous data from each study were recorded. Meta-analysis was performed, if two or more studies with direct head-to-head comparisons were included. This was only possible for the comparison between ASNQ and artemether–lumefantrine (AL) due to limited number of studies in the similar manner. Initially, it was planned to stratify analyses by the targeted malaria parasite (*P. falciparum, Plasmodium vivax*, mixed malaria infection), transmission intensity, brand and dosing of ASNQ. Due to an insufficient number of studies and paucity of data, stratification was not possible.

Sensitivity analysis was not done because of the limited number of studies. Data analysis was performed with RevMan 5·3 [17]. The protocol of the current review is registered with PROSPERO [18].

## Results

**Fig. 1:**
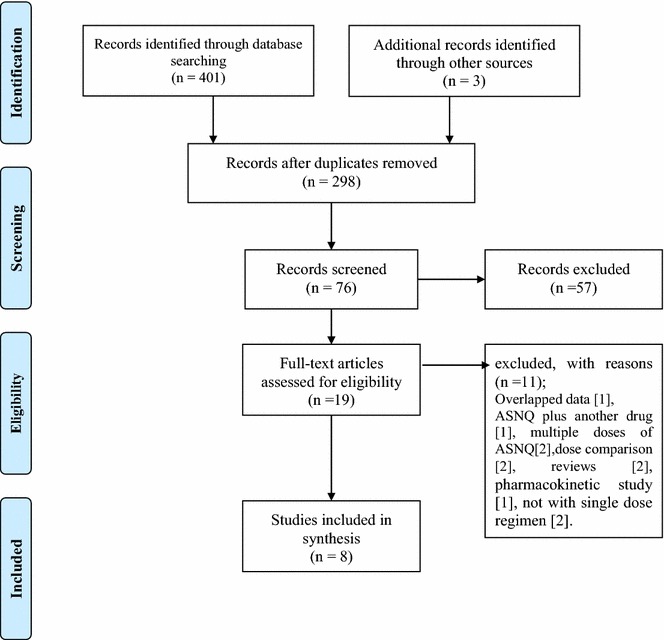
PRISMA flow diagram indicating the process of study selection

The process of study selection is presented in Fig. 1. The initial search yielded 404 citations. Of these, 19 studies that assessed the efficacy and safety of ASNQ were potentially eligible based on titles and abstracts [6, 7, 10, 11, 19–33]. Eleven full-text studies were then removed as they were not appropriate. A final of five RCTs [19–23] and three non-comparative single-arm studies [6, 10, 11] were identified for the current review. A conference abstract [20] was included among these as its complementary information was available in another publication [24]. One study each was published in Chinese [6] and in French [21], while the rest were in English. Four of the five RCTs reported data on the treatment efficacy on day 28 [19, 21–23], while two trials on day 42 [20, 22]. All three single-arm studies reported data at day 28 [6, 10, 11].

Eleven full-text studies were excluded because they (1) overlapped with a study included in this review [7], (2) assessed ASNQ plus another drug [25], (3) had multiple doses of ASNQ [26, 27], (4) were dose comparisons [28, 29], (5) were reviews [24, 30], (6) were a pharmacokinetic study [31], and, (7) not assessed with single dose regimen [32, 33]. A summary of these excluded studies is provided in Additional file 1.

### Characteristics of the included studies

Only five RCTs and three single-arm studies were identified for the current review. The characteristics of the included RCTs are provided in Additional file 2. Of the five RCTs in the current review, one study each was carried out in Benin [21], Indonesia [22], Nigeria [23], Papua New Guinea (PNG) [19], and Uganda [20]. In the present review, single dose ASNQ was compared to AL in three studies [20, 21, 23], CQ plus SP (CQSP) in one study [19] and dihydroartemisinin–piperaquine (DHP) in another study [22]. One single-arm study was done in China [6], Myanmar [11] and Sudan [10] (Table 1). All five RCTs assessed both efficacy and safety outcomes. Most of the RCTs (80 %; 4/5) reported the clinical and laboratory parameters of the participants [19, 21–23]. Less than half of the RCTs (40 %; 2/5) provided PCR-adjusted parasitaemia [21, 22]. Two RCTs [19, 21] and two single-arm studies [10, 11] were funded by the drug manufacturer, Kunming Pharmaceutical Corp (KPC). Two RCTs revealed that the authors had no conflicts of interest [22] and/or the drug manufacturer had no role in the planning, protocol design and execution of the study [19].

**Table 1:** Characteristics of the single-arm studies included in the review

Study author, year of publication [reference]	Country	Main parasite species	Drug schedule	Brand (manufacture)	Dosing	28 day cure rate, n (%)	PCT	FCT
Wang et al., 2003 [6]	China	*P. falciparum*	Single dose^a^	Co-NQ (KPC)	8 Tabs (1000 mg ART + 400 mg NQ)	97/100 (97)	30 ± 8	17.5 ± 12.3
Nour et al., 2013 [10]	Sudan	*P. falciparum*	Single dose	ARCO (KPC)	8 Tabs (1000 mg ART + 400 mg NQ)	120/122 (98.4)	34.8 ± 12.6	12 ± 4.8
Tun et al., 2009 [11]	Myanmar	*P. falciparum*	Single dose	ASNQ (KPC)		52/53 (98.1)	34.6 ± 14.3	18.2 ± 8.6

*ART* artemisin; *ASNQ* artemisinine-naphthoquine; *Co-NQ* artemisinine-naphthoquine; *FCT* fever clearance in hour; *KPC* Kunming Pharmaceutical Corporation, China; *NA* not available; *PCT* parasite clearance in hour; *tabs* tablets

^a^Compared with artemisinine alone or naphthoquine alone

### Methodological quality (Table 2)

Overall, most of the RCTs (80 %, 4/5) included in the current review had ‘low risk of bias’ as they met five of the six domains assessed [19, 21–23]. These clinical trials differed in sample size from 97 [23] to 401 participants [22]. The majority of RCTs in this review (60 %, 3/5) were blinded [20–22] or were open-label studies [19, 22, 23]. This implied both the research investigator and the patient could be aware of the type of treatment that the patient was receiving. Only one study [22] provided the sample size calculation, showing adequate power to detect significant differences between the two treatment arms of the study. Two RCTs were superiority trials, assessing whether ASNQ was more effective than DHP [22] or AL [23]. All these five RCTS reported ITT. While the ITT approach is not ideal, it is considered to be the most appropriate approach for superiority trials since the ITT principle implies a conservative effect on the outcome of the trial [34]. Two clinical trials also reported per protocol analysis [22, 23].

**Table 2:** Review authors ‘judgment on the risk of bias of included trials

Description of domains	Author, publication year [reference no.]				
	Hombhanje et al., 2009 [19]	Rujumba et al., 2010 [20]	Kinde-Gazard et al., 2012 [21]	Tjitra et al., 2012 [22]	Udoh et al., 2014 [23]
Random sequence generation	Yes	Unclear	Yes	Yes	Yes
Allocation concealment	Open label	Unclear	Yes	Open label	Open label
Blinding of outcome assessment	Unclear	Yes	Yes	Yes	Unclear
Incomplete outcome data adequately addressed	Yes	Unclear	Yes	Yes	Yes
Free of suggestion of selective outcome reporting^a^	Yes	Unclear	Yes	Yes	Yes
Addressed other sources of potential bias	Yes	Yes	Yes^b^	Yes	Yes
Sample size calculation	Yes^c^	No	No	Yes	Yes

‘Yes’ indicates ‘low risk of bias; ‘No’ indicates ‘high risk of bias’; ‘Unclear’ indicates ‘uncertain risk of bias’

^a^Included all expected outcomes

^b^Mean parasite counts at baseline are significantly different between the two arms

^c^Described as sufficient sample

### Efficacy

**Fig. 2:**
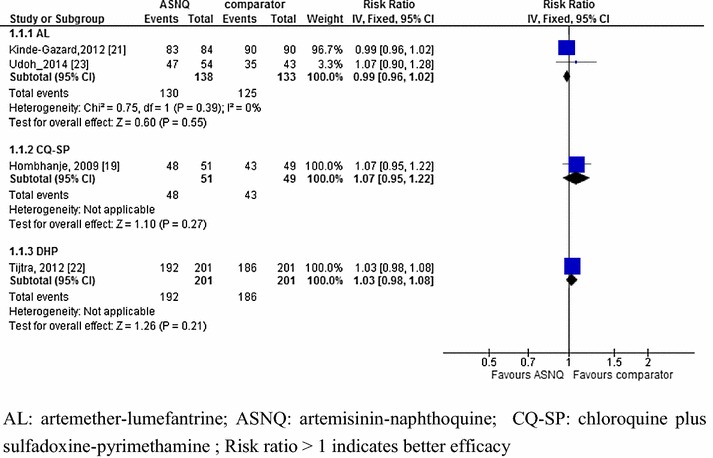
Comparative efficacy between artemisinin–naphthoquine and comparators at day 28

As the RCTs included in the present review did not compare ASNQ with the same anti-malarial drugs, it was difficult to do a pooled analysis. At day 28, a pooled analysis of two RCTs (n = 271) showed a similar efficacy between ASNQ single dose (94.2 %, 130/138) and AL (94 %, 125/133) (RR: 0.99, 95 % CI 0.96–1.02; *I*-square value: 0 %) [21, 23]. Of note, parasitaemia in the Nigeria study was not confirmed by PCR [23]. Also, a comparable efficacy was reported between ASNQ single dose (95.5 %, 192/201) and DHP (92.5 %, 186/201) (RR: 1.03, 95 % CI 0.98–1.08) [22] on PCR-confirmed parasitaemia, and between ASNQ single dose (94.1 %, 48/51) and CQSP (87.8 %, 43/49) on PCR-unconfirmed parasitaemia (RR: 1.07, 95 % CI 0.95–1.22) [19] (Fig. 2).

**Fig. 3:**
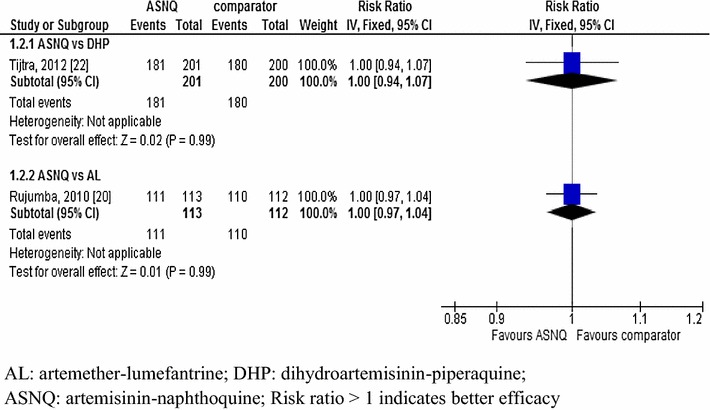
Comparative efficacy between artemisinin–naphthoquine and comparators at day 42

At day 42, there was a similar efficacy between ASNQ (90 %, 181/201) and DHP (90 %, 180/200) on PCR-confirmed parasitaemia (RR: 1.0, 95 % CI 0.94–1.07) [22] and between ASNQ (98.2 %, 111/113) and AL (98.2 %, 110/112) on PCR-confirmed parasitaemia (RR: 1.0, 95 % CI 0.97–1.04) [19] (Fig. 3). Due to a paucity of data, stratify analysis by malaria parasite speciation was not possible. All single-arm studies were assessed on patients with *P. falciparum* malaria.

### Parasite and fever and clearance time (hours)

The mean parasite clearance time (PCT) was comparable between ASNQ (28 ± 11.7) and DHP (25.5 ± 12.2) (MD: 2.5, 95 % CI 0.16–4.64) [22]. The proportion of parasite clearance in the first three days is shown in Table 3. Among adults with malaria, the odds of reduction in parasite clearance within the initial 24 h in the ASNQ group was 1.2 times faster than that in the CQSP [19] or DHP [22]. Almost all parasitaemia was cleared by day 3 [19, 21–23].

**Table 3:** Clearance of blood parasites in the first 3 days

Study author, publication year [reference]	Country	Targeted population	Main parasite species	Drug (n)	Day 1 (odds ratio)	Day 2 (odds ratio)	Day 3 (odds ratio)
Hombhanje et al., 2009 [19]	PNG	Adults	*P. falciparum*	ASNQ (51)	94 %	99.8 %	99.95 %
				CQSP (49)	79 % (1.18)	99.3 % (≈1.0)	99.7 % (≈1.0)
Rujumba et al., 2010 [20]	Uganda	Children	*P. falciparum*	ASNQ	NA	100 %	NA
				AL		100 % (1.0)	
Kinde-Gazard et al., 2012^a^ [21]	Benin	Children	*P. falciparum*	ASNQ (84)	98 %	100 %	99.5 %
				AL (90)	96 % (1.02)	99 % (1.02)	100 % (1.01)
Tjitra et al., 2012^b^ [22]	Indonesia	Adults	*P. falciparum*	ASNQ (201)	70 %	98 %	100 %
				DHP (201)	58 % (1.2)	98 % (1.0)	100 % (1.0)

*PNG* Papua New Guiana

^a^Adapted from Fig. 1 [21]

^b^Adapted from Fig. 4 [22]

Fever clearance time (FCT) was reported in two RCTs; a comparable FCT was shown between ASNQ and DHP (MD: 1.7, 95 % CI −0.05 to 3.45) [20] or ASNQ and CQSP (MD: −3.6, 95 % CI −7.42 to 0.22) [19].

### Gametocytaemia

Only one RCT reported this data. The gametocytaemia by day 3 was reduced from 67.3 to 18.6 % in ASNQ and from 70.3 to 17 % in DHP (*p* > 0.05) [22]. A single-arm study reported that no gametocytes were detected in the blood smears examined [10].

### Adverse events

In all five RCTs, AEs were recorded and compared between treatment groups (i.e., single dose ASNQ group *vs* comparator group). Table 4 provides the list of AEs in the included RCTs. Due to inconsistency in reporting it was difficult to undertake the pooled estimates of AE incidence. The most commonly reported AEs, which were assumed to be drug-related were nausea, vomiting, itchiness, transient deafness, and skin rashes.

**Table 4:** Distribution of adverse events in the studies

Study author, publication year [reference]	Sample size (ASNQ/comparator)	Comparator drug	Study country	ASNQ group	Comparator group
Hombhanje et al., 2009 [19]	51/49	CQ-SP	PNG	Transient deafness: 7.8 %	Transient deafness: 2 %
				Itchiness: 2 %	Itchiness: 4 %
				Skin rash: 2 %	Skin rash: 2 %
				Dark urine: 2 %	Dark urine: 0 %
				Vomiting: 2 %	Vomiting: 4.1 %
Rujumba et al., 2010 [20]	113/112	AL	Uganda	NA	NA
Kinde-Gazard et al., 2012 [21]	84/90	AL	Benin	Nausea: 9.5 %	Nausea: 7.8 %
				Itchiness: 2.3 %	Itchiness: 2.2 %
				Abdominal: pain: 6.3 %	Abdominal: pain: 5.5 %
Tjitra et al., 2012 [22]	201/200	DHP	Indonesia	Nausea: 57 %	Nausea: 54 %
				Headache: 55 %	Headache: 55 %
Udoh et al., 2014 [23]	43/54	AL	Nigeria	Transient maculopapular rash: 0.23 %	None

*AL* arthemether-lumefantrine (Coartem), *ASNQ* artemisinin–naphthoquine single dose, *CQ* chloroquine, *DHP* dihydroartemisinin–piperaquine, *SP* sulphadoxine-pyrimethine, *NA* not available/not mention

### Sensitivity analyses

Per protocol analysis, all patients who were not available (e.g., those withdrawn, lost to follow-up) were removed from the denominator, and this was done in two RCTs [22, 23]. At day 28, one RCT reported a comparable efficacy between ASNQ (87 %, 47/54) and AL (81.4 %, 37/43) on PCR-unconfirmed parasitaemia (*p* = 0.88) [23]. At day 42, another RCT reported a comparable efficacy between ASNQ and DHP (96.3 vs. 97.3 %; *p*: 0.56) on PCR-confirmed parasitaemia [22]. Due to the limited number of studies, sensitivity analysis was not performed. Publication bias was not investigated as the minimum recommended number of studies required to perform this is ten [16].

## Discussion

In order to facilitate the development of treatment policies for the deployment of effective anti-malarial drugs, a systematic monitoring of anti-malarial drug efficacy and drug resistance is needed [5, 34]. The present study reviews the efficacy and safety of the newly co-formulated single dose ASNQ in treating uncomplicated malaria in endemic countries.

### Efficacy

Overall, the findings could provide some evidence that there was a comparable efficacy between ASNQ single dose and comparators such as CQSP, DHP and AL. Moreover, the efficacy estimates of ASNQ were consistent at day 28 and at day 42. Using both day 28 and day 42 in this analysis was relevant because a 28-day follow-up captured the majority of treatment failures with drugs inclusive of artemisinin derivatives, while the longer follow-up for 42 days was optimal for these drugs [35]. RCTs, as well as single-arm studies, showed ≥95 % of therapeutic efficacy, which is a desirable level recommended for a new drug by the WHO [36].

A pooled analysis of studies assessing artesunate–amodiaquine including a single dose regimen in treating uncomplicated malaria documented that rapid *P. falciparum* clearance continued to be achieved in sub-Saharan African patients treated with ACT [37].

### Parasite clearance

The current analysis showed that ASNQ single dose had a property of rapid reduction in parasite biomass within the initial 24 h. In fact, the rate at which treatment clears parasites within the first few days is the most useful practical test for ACT. This is because early response to treatment relies predominantly on the parasite response to artemisinin, independent of whether parasites are later cleared permanently through the combination of longer-lived companion drug and the host’s immune response [38].

### Reduction of gametocytaemia

Although ACT reduced the density of gametocytaemia and the proportion of infected mosquitoes, sub-microscopic levels of gametocytes (which were present in a significant number of patients after treatment) appeared to be sufficient to drive post-treatment transmission [39]. The detection of gametocytes by microscopy represents only the ‘tip of the iceberg’. Therefore, relying only on gametocyte carriage as an indicator can result in an overestimation of the effects of treatment regimens [19].

### Safety measure

One of the key elements in any drug development and evaluation is the issue of safety for the population for whom the drug is intended [25]. The incidence of AEs in the included studies was presented in various ways due to inconsistency in the symptoms reported or actual variation in the symptoms attributed to the drug. This created difficulties in pooling the results. A study in Nigeria [28] compared three ASNQ treatment regimens in ≥15-year-old children (n = 121), comparing four tablets as a single dose (700 mg, group A), eight tablets as a double dose (1400 mg, group B) and eight tablets as a single dose (1400 mg, group C). Treatment success was not significantly different among these three groups. The occurrence of a blister on the lips was reported in a patient receiving the double dose of ASNQ, but not in patients receiving the single dose [29]. This can reflect potential problems of tolerability with the double dose. Further careful monitoring of ASNQ-related AE and dose response effects are needed. ASNQ-related SAEs were not reported in any of the studies included in the current review.

### Schedule of therapy

A study assessing a dose comparison of ASNQ in children with malaria showed similar efficacy and tolerability between single dose with water/with milk and twice daily dose regimens, indicating single dosing can be expected to improve better compliance [28]. A shorter regimen, such as single dose coformulated drug (ASNQ in this case), is usually better than a three-day regimen for ideal compliance (to the treatment schedule) [11]. Although any of the current regimens could be given for 1 day only the three-day regimens were considered. In the three-day regimen, the artemisinin component is present in the body for a short enough time to cover only two asexual parasite life cycles, except in the case of *Plasmodium malariae*. In each cycle, artemisinin and its derivatives would reduce parasite biomass by a factor of ~10,000 [19, 40].

A dose range study in PNG showed that a lower single ASNQ dose was associated with relatively frequent recurrence of *P. vivax* infections [28]. The recommended ACT (for instance, AL, artesunate-mefloquine) follows three-day treatment regimens. A clinical trial showed that the three-day ASNQ was non-inferior to AL for the treatment of uncomplicated falciparum malaria among young children in PNG and had greater efficacy than AL against vivax malaria [32, 33].

Any anti-malarial regimen must have robust evidence of both optimal efficacy for patient survival and an ability to reduce the potential for drug resistance [19]. The findings of this review, based on available data, showed evidence on the efficacy and tolerability of the ASNQ single dose. Continuous monitoring of parasite resistance to this ASNQ is still needed.

### Study limitations

There are limitations to the present study. A small number of studies with small samples on the assessment of the ASNQ single dose were identified for the present review. In order to demonstrate the non-inferiority (by a margin of 5 %) of an alternative treatment (ASNQ in this case) to a current treatment known to be 95 % effective, at least 299 patients would be necessary in each study arm, with a one-sided test that has a statistical power of 80 % and a significance level of 2.5 % [41]. Using the best efficacy data (e.g., 100 % in ASNQ and 98.7 % in comparator) and the same 5 % non-inferiority margin, type 1 error probability and power as in the original sample size estimates, the total of 52 participants would be required to show non-inferiority for this primary endpoint [32]. If so, all five RCTs [19–23] seemed to be sufficient to define the efficacy in two groups. However, the PNG study [19] had reported efficacy in the ASNQ (94 %) and comparator (80 %), hence, the required sample size was 348, which was about 3.5 times higher than the reported total of 100 participants.

The PCR-confirmation of parasites was done in only two RCTs in the current review [21, 22], hence, the chance of diagnostic bias is a concern. This was more prominent in areas where *P. falciparum* and *P. vivax* co-exist. For instance, the efficacy of ASNQ and CQSP were comparable in the PNG study where *P. falciparum* and *P. vivax* are equally important [19]. Thus, *P. vivax* was likely to be missed in PCR-uncorrected parasitaemia. This could be more pronounced in the CQSP group as parasite resistance to this combination drug is already confirmed in PNG [19]. The efficacy reported in the PNG study, therefore, needs to be interpreted in the light of these important biases. Moreover, PCR is useful for classification of re-infection or recrudescence. Hence, misclassification bias is a concern in the absence of PCR confirmation.

Three RCTs in this review were open-label trials [19, 22, 23]. As both the research investigator and the patient could be aware of the type of treatment this might lead to reporting bias of AEs in open-label trials [42]. Furthermore, true anti-malarial drug resistance may not be the same as treatment failure. If the administration of anti-malarial drug does not reach a sufficient blood concentration level, it will cause an inability to clear parasites in that malaria episode [1, 43]. Due to a lack of information on blood concentrations of the drugs administered, the current findings should be interpreted with a high degree of caution.

Due to limited data, the current review is unable to provide evidence for different age groups or different *Plasmodium* species. All single-arm studies were conducted on patients having *P. falciparum* [6, 10, 11]. It is likely that studies which were not published in the peer-reviewed literature might have been missed. However, not being in peer-reviewed literature raises questions about the quality of the study and its evidence. Another concern is that an assessment of therapeutic efficacy between ASNQ and CQSP [19] needs caution as CQ is a relatively old drug with known resistance by *P. falciparum*.

### Public health implications

Based on available data, the current analysis shows comparable efficacy and safety profiles of the single dose ASNQ. Single dose ASNQ can improve treatment compliance and is simple and practicable. This is because this single dose can be delivered as directly observed treatment, immediately after confirmation of malaria [11]. Should mass drug administration be required in an outbreak of malaria, the (ASNQ) single dose could be a key element in community deployment of ACT. However, a single dose (even with a repeated dose on day 2) is insufficient as it can expose only one asexual cycle to the arteminisin component. Although a high cure rate could be attained from an ACT containing an effective partner drug, this will not provide sufficient protection from drug resistance [44]. In fact, malaria parasites will be eliminated, and the patient cured, if the plasma concentration of the free anti-malarial drug (ASNQ in this case) still exceeds the concentrations required to maintain the parasite multiplication rate below one (the minimum inhibitory concentration) [45]. The risk of the development of de novo resistance is increased with the duration of time the dividing asexual parasites are exposed to drugs. This makes the long-term risk of developing resistance a concern for single dose ACT [22]. As the ultimate goal of combination therapy is to prevent resistance developing, the usefulness of single dose ASNQ for treating uncomplicated malaria in endemic countries needs further evaluation.

The relatively long terminal half-life and wide therapeutic index of NQ [29] could contribute to better efficacy in three-day ASNQ regimen. This is because NQ can suppress the re-appearance of both *P. falciparum* and *P. vivax* for a longer period than piperaquine and, in particular lumefantrine. This is because the peak plasma concentration of benflumetol (i.e., the chemical name for lumefantrine) is attained slowly (8 h plus a 2-h absorption lag time), and the elimination half-life is estimated to be 4.5 days [46]. Although the resistance mechanisms of NQ are unknown, there is a potential for it to develop cross-resistance between NQ and CQ (both are 4-aminoquinoline derivatives) and a slight cross-resistance between NQ and artemisinin, lumefantrine or pyrimethamine (these drugs are unrelated to NQ both chemically and in terms of mechanism of action). The decreasing efficacy of amodiaquine single-agent treatment have been reported in some sub-Saharan African settings. If artemisinin resistance occurs there, it is expected to result soon in treatment failures, particularly in semi-immune populations such as children in high-transmission areas or patients of all ages in low transmission areas [37]. There is no study on single dose ASNQ with special groups such as pregnant women and infants and these are still needed.

## Conclusions

The present review provide some evidence to support the comparable efficacy and safety of the single dose ASNQ compared to other comparator anti-malarial drugs in treating uncomplicated malaria. Larger, adequately powered, well-designed studies are recommended to substantiate the efficacy and safety in different populations and in different epidemiological settings. As the potential evolution of drug resistance is a great concern and this cannot be addressed in a short-term study, the use of single dose ASNQ needs further evaluation.

## Additional files

10.1186/s12936-015-0919-5 Summary of the excluded studies.
10.1186/s12936-015-0919-5 Characteristics of the included studies.

## Abbreviations

ACT: artemisinin-based combination therapy
AE: adverse events
AL: artemether–lumefantrine
ASNQ: artemisinin–naphthoquine
CI: confidence interval
CQ: chloroquine
CQSP: chloroquine plus sulfadoxine-pyrimethamine
DHP: dihydroartemisinin–piperaquine
FCT: fever clearance time
HR: hazards ratio
ITT: intention-to-treat
MD: mean difference
NQ: naphthoquine phosphate
OR: odds ratio
PCT: parasite clearance time
PICOS: participants/population, Intervention, comparator(s), outcome(s), study design
PNG: Papua New Guinea
PRISMA: preferred reporting items for systematic reviews and meta-analyses
RR: relative risk
SAE: serious adverse events
SD: standard deviation
WHO: World Health Organization
