# Supplementary material for: A systematic review of the efficacy of a single dose artemisinin–naphthoquine in treating uncomplicated malaria
Source: Malar J. 2015 Oct 6;14:392. doi: 10.1186/s12936-015-0919-5 (PMC4596557; doi:10.1186/s12936-015-0919-5)
Supplement: Supplementary file 1 — 10.1186/s12936-015-0919-5 Summary of the excluded studies. [file 12936_2015_919_MOESM1_ESM.doc]

Additional File 2

Summary of the excluded studies

| Study, [ref] | Country | assessments | Key finding |
| --- | --- | --- | --- |
| Wang, [7] | China | NQ (n, 30) vs ART( n, 30) | Cure rate : NQ: 96.7% vs ART 73.3% |
| Hombhanje, 2010 [24] | - | Overview | SD ASNQ is equally effective and safe as the 3 D treatment with AL and other existing ACTs. |
| Krudsood, [25] | Thailand | DNP vs AL | Efficacy, at D 28: DNP 99% (80/81) vs AL 97% (33/34) |
| Liu, 2013 [26] | China | An open-label, non-inferiority trial on aged 5-16 yr: 9%, > 16 yr: 91%;  ASNQ 3 daily dose vs CQ-PQ;  ASNQ (n, 127) vs CQ-PQ(n, 128) | Efficacy, at D 42: ASNQ 98.4% (125/127) vs CQ-PQ 96.1% (123/128) |
| Tour, [27] | Côte d'Ivoire | *P.f*, halo endemic areas,  2 doses on 1day  ASNQ (n, 62) vs AL(n, 61) | Efficacy, at D 28: ASNQ 100% (62/62) vs AL 98.4% (60/61),  Parasite clearance: by D 1: 71% in ASNQ vs 69% in AL;  by D 3, 100% in both group |
| Benjamin, 2012 [28] | PNG | Dose comparison on 5-12 yr children;  ASNQ given to 3 groups  Gr 1: 2-4 SD (with water)  Gr 2: 3-6 tabs (with milk);  Gr 3:3-6 tabs twice (with water) | Efficacy,  Gr 1: 14/14, 93% (70-98%) at D28 & at D42;  Gr 2: 17/17, 100% at D42;  Gr 3:16/16, 100% at D42. |
| Meremikwu, 2012 [29] | Nigeria | Dose comparison on ≥ 15 yr children;  ASNQ 3 daily dose vs AL  Gr 1: 700 mg (4 tab) SD x 3D;  Gr 2:700 mg twice per D x 3D;  Gr3:1,400 mg (8 tab) SD x 3D | Efficacy, at D 28, over all: 88.9%  Gr 1: 85.3%;  Gr 2:93.1%;  Gr 3:88.9% |
| Isba, 2015[30] | - | Review  4 RCTs, SD and multiple doses trials | Both ASNQ and comparators (AL, DHP) had low treatment failure. |
| Betty, [31] | PNG | pharmacokinetic study  Gr 1: SD ASNQ with water ( 13),  Gr 2: SD ASNQ with water (17),  Gr 3: twice daily ASNQ (16)  Weight base dosages. | Gr 1: 54% (7/13) recurrent (+) during 42 D follow up;  Gr 3: 0% (0/13) recurrent (+) during 42 D follow up;  pharmacokinetic characterization justify using the SD ASNQ for 3 D. |
| Laman,2014 [32] | PNG | An open-label, non-inferiority trial on aged 1/2-5 yr children;  ASNQ 3 daily dose vs AL | ASNQ 3 D therapy was non-inferiority to AL among under 5 yr children.  PCR-confirmed efficacy for *P. f*, at D 28: ASNQ 100% (96/96) vs AL 98.9% (93/94);  at D42: ASNQ 100% (94/94) vs AL 97.8% (90/92);  PCR-unconfirmed efficacy for *P. v*, at D 28: ASNQ 100% (96/96) vs AL 98.9% (93/94);  at D42: ASNQ 100% (94/94) vs AL 97.8% (90/92) |
| Laman, 2015 [33] | PNG | Post-treatment assessment of earlier study on ASNQ 3 daily dose vs AL | Fewer clinical malaria within 6 mths after Rx in ASNQ (11.2% 10/89) than in AL (23% 20/87) on children aged ½ - 5yr;  Median time to 1^st^ malaria illness was longer in ASNQ (64, 50-146 D) vs AL (116, 77-130 D). |

ASNQ: artemisinin-naphthoquine;AL:artemether-lumefantrine; ART: Artemisin; D: day; DNP: dihydroartemisinin-napthoquine-trimethoprim; Gr: group; n = number; NQ: naphthoquine ; PNG: Papua New Guiana; SD: single dose;
