# Supplementary material for: A systematic review of the efficacy of a single dose artemisinin–naphthoquine in treating uncomplicated malaria
Source: Malar J. 2015 Oct 6;14:392. doi: 10.1186/s12936-015-0919-5 (PMC4596557; doi:10.1186/s12936-015-0919-5)
Supplement: Supplementary file 2 — 10.1186/s12936-015-0919-5 Characteristics of the included studies. [file 12936_2015_919_MOESM2_ESM.doc]

Additional file 3

Characteristics of the included studies

| Author, publication year [Reference no.] | Study country | Comparator | Sample size  (ASNQ/ comparator) | Targeted parasite | Age ^a^ | male | Brand  (manufacturer) | dosage | supervised | PCR confirmation | Funding source |
| --- | --- | --- | --- | --- | --- | --- | --- | --- | --- | --- | --- |
| Hombhanje et al., 2009 [19] | PNG | CQ-SP | 51/49 | *P.f* | 27.5 (14-60) | 53% | ARCO,  (KPC) | single dose on a single day  (AS: 1000mg + NQ: 400 mg) | yes | no | KPC |
| Rujumba et al., 2010 [20] | Uganda | AL | 113/112 | NA | 4m-16m | NA | ARCO,  (KPC) | single dose | NA | NA |  |
| Kinde-Gazard et al., 2012 [21] | Benin | AL | 84/90 | *P.f* | 4.8m  (6m-13.8yr) | 44% | ARCO,  (KPC) | single dose on a single day  (AS: 125mg + NQ: 50 mg) | yes | yes |  |
| Tjitra et al., 2012 [25] | Indonesia | DHP | 201/200 | *P.f, P. v,mixed* | 27.6 (±10.8) | 90% | ARCO,  (KPC) |  | yes | yes | KPC |
| Udoh et al, 2014 [23] | Nigeria | AL | 54/43 | *P.f* | 5-14 | NA | ARCO,  (KPC) | single dose on a single day | yes | no | KPC |

^a^ median (range) or mean ± SD; ^b^ registered no. in China; ^c^ information on the manufacture not indicated; ^d^ given at the clinic and assumed supervised; KPC: Kunming Pharmaceuticals, Kunming, China; AS: artemisinin; NQ: naphthoquine; m:months; yr: year; DH: dihydroartemisinin; TP: trimethoprim; DHP: dihydroartemisinin- piperaquine; *P.f*: *Plasmodium falciparum; P.v*: *Plasmodium vivax*; DNP: compound naphthoquine, developed in China; AL:arthemether-lumefantrine (Coartem); NA: not available/not mention; CQ: Chloroquine; SP: sulphadoxine-pyrimethine; PQ:primaquine ;
